# Depressive symptoms in stroke patients treated and non-treated with intravenous thrombolytic therapy: a 1-year follow-up study

**DOI:** 10.1007/s00415-018-8938-0

**Published:** 2018-06-18

**Authors:** Barbara Grabowska-Fudala, Krystyna Jaracz, Krystyna Górna, Izabela Miechowicz, Izabela Wojtasz, Jan Jaracz, Radosław Kaźmierski

**Affiliations:** 1 Department of Neurological Nursing, Poznan University of Medical Sciences, Smoluchowski 11 Str., 61-170 Poznan, Poland; 2 Department of Psychiatric Nursing, Poznan University of Medical Sciences, Poznan, Poland; 3 Department of Computer Science and Statistics, Poznan University of Medical Sciences, Poznan, Poland; 4 Department of Neurology and Cerebrovascular Disorders, Poznan University of Medical Sciences, L. Bierkowski Hospital, Poznan, Poland; 5 Department of Adult Psychiatry, Poznan University of Medical Sciences, Poznan, Poland

**Keywords:** Stroke, Thrombolysis, Depression, Posttraumatic stress symptoms

## Abstract

**Introduction:**

This is a prospective study, first to compare the frequency of depressive symptoms in stroke survivors treated, and non-treated, with intravenous thrombolysis and second, to explore relationships between post-stroke depression (PSD) and stroke treatment modalities, taking into account other possible determinants of PSD, including post-traumatic stress symptoms.

**Methods:**

Groups of 73 thrombolysed and 73 non-thrombolysed patients matched for age and gender were examined at 3 and 12 months after discharge. PSD was assessed using the Beck Depression Inventory. Post-traumatic stress symptoms (PTSS), disability and social support were assessed with the Impact of Event Scale-Revised, the Barthel Index and the Berlin Social Support Scale.

**Results:**

At 3 months, PSD was present in 23.3% of the thrombolysed and 31.5% in the non-thrombolysed groups (*p* = 0.265). At 12 months, the frequencies were 29.2 and 20.6% (*p* = 0.229). Logistic regression of the combined group of thrombolysed and non-thrombolysed patients indicated that at 3 months, the adjusted predictors of PSD were disability (OR 24.35), presence of PTSS (OR 9.32), low social support (OR 3.68) and non-thrombolytic treatment (OR 3.19). At 12 months, the predictors were disability (OR 15.78) and low education (OR 3.61).

**Limitations:**

The use of a questionnaire for the detection of depression, the relatively small sample size and a significant drop-out rate could limit the interpretation of these results.

**Conclusions:**

(1) Thrombolysed and non-thrombolysed stroke survivors had similar frequency of depressive symptoms although the thrombolysed patients had more severe neurological deficits in the acute phase. It can be assumed that if thrombolysis had not been used, depressive symptoms would have been more frequent. (2) Lack of the rt-PA treatment was associated with three-time greater odds of screening for PSD at 3 months post-stroke, after adjustment for other PSD correlates. (3) Therefore, thrombolytic therapy seems to have a positive, but indirect, effect on patients’ mood, especially in the first months after stroke. (4) All stroke patients, irrespective of the method of treatment, should be monitored for the presence of depression.

## Introduction

Intravenous (i.v.) thrombolytic therapy with recombinant tissue plasminogen activator (rt-PA) was first registered in the United States in 1996 [1]. Since that time, efficacy studies on thrombolysis have demonstrated its positive impact on functional recovery by reducing the risk of physical disability at 3–18 months following the treatment [2, 3]. The mechanism of the favourable effect of rt-PA is explained by early recanalization of occluded arteries and, consequently, the restoration of regional perfusion and a reduction in the size of the brain infarct [4].

In contrast to the well-recognized link between thrombolysis and physical independence [5], its association with psychological well-being remains largely unexplored.

There is broad agreement that neuropsychiatric disorders, especially post-stroke depression (PSD), are severe complications of stroke. It has been found that PSD significantly reduces the quality of life and increases the risk of mortality in the first years after a stroke [6–8]. According to a recent systematic review, PSD affects 29% of patients at any time point after stroke [6].

Several factors are related to PSD, but among the most consistently reported in the literature is physical disability resulting from stroke [8–10]. Therefore, given the effect of thrombolysis on the functional outcomes described above and the significance of the problem, that is PSD, it seems important and reasonable to investigate PSD in patients receiving rt-PA, to determine whether these patients are also less likely to develop depressive symptoms [11, 12].

To date, only a few researchers have addressed this issue, and their studies have shown that the frequency of depression does not differ significantly between rt-PA-treated and -non-treated patients at 12 [13] and 18 months after stroke [2]. Recently, the authors of the Telemedical Project for Integrative Stroke Care (TEMPiS) study [14] proposed a possible explanation for the observed findings, namely the role of the psychological response to the traumatic event, that is, an acute stroke, together with peri-thrombolysis procedures. However, the lack of a comparison group of non-thrombolysed patients did not allow the authors to check this hypothesis.

The aims of the present study were first to compare the frequency of depressive symptoms in ischaemic stroke survivors treated and non-treated with rt-PA and second, to explore relationships between post-stroke depression and stroke treatment modalities, taking into account other possible determinants of PSD, including post-traumatic stress symptoms.

## Methods

### Study participants

This study is part of a larger research project on recovery from stroke and includes patients consecutively treated with i.v. rt-PA in the University Department of Neurology and Cerebrovascular Disorders in Poznan in the years 2012–2014. Our previous results have been described elsewhere [15]. Thrombolysis was performed within 4.5 h from the onset, according to the European guidelines [16]. Our research inclusion criteria required that the patients had an ischaemic stroke confirmed by brain imaging (computed tomography or magnetic resonance), were at least 18 years old, had no other chronic or serious health conditions, except for stroke risk factors, lived in the Wielkopolska region of Poland and gave their written, informed consent.

Initially, 114 rt-PA-treated patients were enrolled, of whom 41 (35.9%) dropped out: 14 died, 9 were transferred to another ward, 3 were discharged to a nursing home, 1 had severe cognitive problems, 5 refused to participate and 9 were lost to follow-up after discharge. The final thrombolysed group, therefore, consisted of 73 participants.

Patients who were not thrombolysed, mainly due to late hospital arrival, constituted a gender/age-matched group for comparison with the thrombolysed group. Of the 127 no-rt-PA treated initially included, 54 (42.5%) dropped out for the following reasons: death (*n* = 18), transfer to another ward (*n* = 3), discharge to a nursing home (*n* = 2), refusal of participation (*n* = 18) and lost to follow-up (*n* = 13), resulting in a final matched group of 73 patients.

The drop-out analysis showed that in both the rt-PA-treated and the -non-treated groups the patients who dropped out, in comparison to those who remained in the study, had a more severe stroke at baseline (medians NIHSS 15 vs. 8.0, *p* < 0.001, in the thrombolysed group; medians NIHSS 6.0 vs. 4.0, *p* < 0.026, in the non-thrombolysed group) and were older (mean age 71.1 years vs. 66.3, *p* = 0.039, in the thrombolysed group; mean age 72.7 years vs. 66.8, *p* = 0.003, in the non-thrombolysed group). However, the gender of the participants and non-participants in both the thrombolysed and non-thrombolysed groups was equivalent (*p* > 0.05).

The research protocol was approved by the local ethics committee.

### Study design and measures

#### Study design

The patients’ follow-up data were collected upon admission (Time 0), at discharge (Time 1), at 3 months (Time 2) and 12 months after hospital discharge (Time 3). Data concerning T0 and T1 were collected during hospitalization and included socio-demographic and clinical characteristics. The socio-demographic characteristics included age, gender, education and living arrangement. Clinical data included stroke severity at admission and discharge, lesion location, comorbidities and functional status at discharge. Data for each patient were obtained from the medical records and through direct patients’ assessment. Time 2 and Time 3 data were collected via a face-to-face at-home interview and an assessment was made which included stroke severity, functional status, depressive symptoms, post-stroke post-traumatic stress symptoms and social support.

#### Measurement tools

Stroke severity was determined according to the National Institutes of Health Stroke Scales (NIHSS) [17]. The NIHSS score ranges from 0 to 42, with higher values indicating a more severe neurological deficit. In this study, the severity of stroke was classified as mild (0–8 points on the NIHSS scale) and moderate/severe (≥ 9 points) [18]. Functional status was assessed with the modified Barthel Index (BI) developed by Collin and Wade [19]. This scale measures capacity to perform ten basic activities of daily living and is scored from 0 to 20, where scores of 0–14 indicate severe or moderate disability and 15–20 represent mild or no disability [20]. Depressive symptoms were evaluated with the Beck Depression Inventory (BDI) (Polish version) [21, 22]. The BDI consists of 21 items, with a total score ranging from 0 to 63, where a higher score indicates more severe depressive symptoms. A cut-off point at 18 was used to distinguish between the presence and absence of a clinically relevant depression [21]. Posttraumatic stress symptoms (PTSS) linked to stroke were assessed with the Impact of Event Scale-Revised (IES-R, Polish version) developed by Weiss et al. [23, 24]. This comprises 22 items and yields a total score of between 0 and 88. A score of 33 or higher is regarded as the existence of distress symptoms [25]. The IES-R has already been used in various illness populations, including stroke patients [26, 27] and has been shown to have good psychometric properties [24].

Perceived social support was evaluated using the Berlin Perceived Social Support Scale (BSSS, Polish version) [28, 29]. The BSSS consists of eight items, each rated on a four-point Likert scale. The total score is calculated as a mean of the item scores, and ranges from 1 to 4, with higher values reflecting greater levels of support. Patients who scored less than the median of the total score were classified as those with low social support, and patients who scored equal and higher than the median were regarded as those with high social support.

### Statistical analysis

The descriptive statistics are presented as the means ± standard deviations (SD), medians or frequencies, as appropriate. Associations between two qualitative variables were analysed using the Chi-squared test or the Fisher’s exact test depending on the group size. Between-group comparisons were performed with the Student *t* test or the Mann–Whitney *U* test. The McNemar’s test was applied to analyse changes in proportions between 3 and 12 months post-stroke. Multiple logistic regression analysis was conducted to identify independent associations of socio-demographic and clinical variables with depression status at 3 and 12 months after stroke. In our regression models, we first entered candidate determinants, with a significance level < 0.25 in bivariate analyses in at least one of the two follow-up assessments [30]. Because of high correlations close to 0.80, between the NIHSS and BI scores at the 3- and 12-month follow-ups, as having the potential for multicollinearity, only the BI was selected for the analyses. Next, we developed a final model using the backward-elimination procedure, retaining only those variables that were significant at *p* < 0.05. The analyses were performed using Statistica 12 (StatSoft, Inc.) and SPSS (SPSS Inc., Chicago, IL, USA) software.

## Results

### Socio-demographic and clinical characteristics of the patients

The thrombolysed group included 45 men and 28 women, with a mean age of 66.27 years (± SD 11.4; range 31–91), in most cases with a primary or basic vocational education (60.1%), and living with a family (89.0%). The non-thrombolysed group also contained 45 men and 28 women of a similar age (mean 66.84 ± SD 11.3; range 32–89), education and living arrangement. Upon admission (T0), the two groups did not differ significantly concerning stroke location and comorbidities. The thrombolysed group had more severe stroke symptoms (median NIHSS 8.0 vs. 4.0 for the non-thrombolysed group, *p* < 0.001) and a higher percentage of subjects with moderate/severe stroke compared to the non-thrombolysed patients (42.5 vs. 19.8%, *p* = 0.002). At discharge, the two groups were similar in terms of functional status and the proportions of moderate/severe stroke, although the thrombolysed patients still had more severe stroke symptoms (median NIHSS 4.0 vs. 2.0, *p* = 0.011). The decrease in the median NIHSS score between T1 and T0 was greater in the thrombolysed (median NIHSS 8.0 vs. 4.0) than in the non-thrombolysed patients (median NIHSS 4.0 vs. 2.0). Similarly, the decrease in the proportion of patients with moderate/severe stroke was greater in the thrombolysed group than in the non-thrombolysed group (17 vs. 6%) (Table 1).

**Table 1:** Demographic and clinical characteristics at hospital admission and discharge

Characteristics	Thrombolysed group	Non-thrombolysed group	*p*
Gender, *n* (%)			1.000
Male	45 (61.6)	45 (61.6)	
Female	28 (38.4)	28 (38.4)	
Age, mean (± SD)	66.3 (11.39)	66.8 (11.28)	0.765
Education, *n* (%)			0.614
Primary or basic vocational school	44 (60.3)	41 (56.2)	
Secondary or high school	29 (39.7)	32 (43.8)	
Living arrangement			0.461
Alone	8 (11.0)	11 (15.1)	
With family	65 (89.0)	62 (84.9)	
Stroke severity at admission			
NIHSS score, median (IQR)	8.0 (5–13)	4.0 (3–7)	< 0.001
NIHSS score of 9 or higher, *n* (%)	31 (42.5)	14 (19.8)	0.002
Stroke severity at discharge (IQR)			
NIHSS score, median (IQR)	4.0 (2–8)	2.0 (1–5)	0.011
NIHSS score of 9 or higher, *n* (%)	18 (24.7)	10 (13.7)	0.093
Stroke risk factors, *n* (%)			
Hypertension	63 (86.3)	59 (80.8)	0.371
Diabetes	16 (21.9)	20 (27.4)	0.442
Obesity	12 (16.4)	9 (12.3)	0.479
Hypercholesterolemia	15 (21.0)	20 (27.0)	0.332
Atrial fibrillation	21 (28.8)	16 (21.9)	0.341
Ischaemic heart disease	11 (15.1)	9 (12.3)	0.630
Stroke location, *n* (%)			0.148
Left hemisphere	45 (61.6)	35 (47.9)	
Right hemisphere	23 (31.5)	27 (37.0)	
Bilateral and other	5 (6.9)	11 (15.1)	
Functional status at discharge, *n* (%)			
BI score, median (IQR)	18.0 (9–20)	19 (13–20)	0.402
BI score of 14 or less, *n* (%)	28 (38.4)	23 (31.5)	0.385

*SD* standard deviation, *IQR* interquartile range, *NIHSS* National Institutes of Health Stroke Scale, *BI* Barthel Index

### Frequency of depressive symptoms at 3 and 12 months

A personal history of depression was documented in three survivors (two in the thrombolysed and one in the non-thrombolysed group). These patients were not excluded from the study. At the 3-month follow-up, 17 (23.3%) patients of the thrombolysed and 23 (31.5%) of the non-thrombolysed group exhibited depressive symptoms according to the BDI, and no significant difference between these proportions was found (*p* = 0.265). Also, severity of symptoms did not differ significantly (median BDI = 12, IQR 7–17 vs. median = 13, IQR 6–20; *p* = 0.976, respectively). At 12 months, depressive mood was present in 21 (29.2%) of the rt-PA-treated and in 15 (20.6%) of the -non-treated patients. Again, no significant difference was seen between the two groups (*p* = 0.229). The median values of the BDI were 10 (IQR 3–19) and 10 (IQR 4–16) *p* = 0.935. Nevertheless, when the odds ratios are taken into account (instead of the *p* values alone), a trend in favour of the thrombolysed patients could be seen at 3 months (unadjusted OR for depression = 0.66) and the opposite can be observed at 12 months (OR for depression = 1.57).

Additional analyses performed after exclusion of the three patients with a personal history of depression showed similar results in relation to the proportions of patients with depressive symptoms in the thrombolysed and non-thrombolysed groups, at both the 3 (*p* = 0.278, OR = 0.66) and 12 month-follow-ups (*p* = 0.220, OR = 1.62).

The majority of patients were stable regarding their depressive status between 3 and 12 months post-stroke, in both the thrombolysed (83.3%) (*p* = 0.388) and non-thrombolysed (76.4%) (*p* = 0.098) groups. It should be added that, at the 3*-* and 12*-*month follow*-*ups, the groups did not differ significantly with respect to their neurological status (*p* = 0.826, *p* = 0.079, respectively) and functional status (*p* = 0.829, *p* = 0.967, respectively).

### Factors associated with depressive symptoms at 3 and 6 months after stroke

As there were no significant differences between the thrombolysed and non-thrombolysed patients concerning their depressive status, the two groups were combined to explore further the relationships between depressive symptoms and thrombolytic therapy in the presence of other possible correlates of depression, bearing in mind the OR values obtained (see section above). These analyses showed that at the 3-month follow-up, 10 (6.8%) patients had moderate/severe stroke symptoms and 22 (15%) demonstrated moderate/severe disability, 36 (25.2%) experienced post-traumatic stress symptoms and 51 (35%) reported low perceived social support. Bivariate analyses comparing the depressed and non-depressed patients showed that post-stroke depression was significantly associated with more severe stroke symptoms at follow-up (*p* < 0.001), disability (*p* < 0.001), the presence of post-traumatic stress symptoms (*p* < 0.001) and low social support (*p* < 0.019) at *p* < 0.05.

At 12 months follow-up, 3 (2%) patients had moderate/severe stroke symptoms, 15 (10%) moderate/severe functional disability, 37 (25.3%) suffered from post-traumatic stress symptoms and 57 (39%) declared low social support. Stroke symptom severity (*p* < 0.014), the level of disability (*p* < 0.001) and education (*p* = 0.001) were the factors significantly associated with the depressive status at *p* < 0.05. Detailed results are presented in Table 2.

**Table 2:** Factors associated with depressive status at 3 and 12 months post-stroke

	Depressive status at 3 months *n* = 146			Depressive status at 12 months *n* = 145*		
	Depression (*n* = 40)	No depression (*n* = 106)	*p*	Depression (*n* = 36)	No depression (*n* = 109)	*p*
Gender, female, *n* (%)	19 (47.5)	37 (34.9)	0.163	13 (36.1)	43 (39.5)	0.721
Age years, mean (± SD)	67.9 (10.7)	66.0 (11.5)	0.397	65.9 (10.2)	66.3 (11.6)	0.722
Education, primary or basic vocational, *n* (%)	27 (67.5)	58 (54.7)	0.162	29 (80.6)	55 (50.1)	0.001
Living arrangement, alone, *n* (%)	6 (15.0)	13 (12.3)	0.661	5 (13.9)	13 (11.9)	0.773
Left hemisphere stroke, *n* (%)	22 (55.0)	58 (54.7)	0.810	20 (55.6)	58 (53.2)	0.807
Hypertension, *n* (%)	32 (80.0)	90 (84.9)	0.476	29 (80.6)	92 (84.4)	0.590
Diabetes, *n* (%)	13 (32.5)	23 (21.7)	0.177	12 (33.3)	23 (21.1)	0.137
Obesity, *n* (%)	6 (15.0)	15 (14.2)	0.896	4 (11.1)	17 (15.6)	0.596
Hypercholesterolemia, *n* (%)	7 (17.5)	28 (26.4)	0.260	8 (22.2)	27 (24.8)	0.757
Atrial fibrillation, *n* (%)	12 (30.0)	25 (23.6)	0.427	10 (27.8)	26 (23.9)	0.637
Ischaemic heart disease, *n* (%)	7 (17.5)	13 (12.3)	0.412	7 (19.4)	13 (11.9)	0.257
NIHSS at admission ≥ 9, *n* (%)	17 (42.5)	28 (26.4)	0.060	20 (55.6)	28 (27.7)	0.033
NIHSS at follow-ups ≥ 9, *n* (%)	8 (20.0)	2 (2.0)	< 0.001	3 (8.3)	0 (0.0)	< 0.014
BI at follow-ups ≤ 14, *n* (%)	15 (37.5)	7 (6.6)	< 0.001	12 (33.3)	3 (2.8)	< 0.001
IES > 33, *n* (%)	18 (45.0)	18 (17.0)	< 0.001	11 (30.6)	26 (23.9)	0.423
BSSS < median, *n* (%)	20 (50.0)	31 (29.3)	0.019	19 (52.8)	38 (34.9)	0.056
Stroke treatment, no-rt-PA, *n* (%)	23 (57.5)	50 (47.2)	0.265	15 (41.7)	58 (53.2)	0.229

Bivariate analysis

*NIHSS* National Institutes of Health Stroke Score, *BI* Barthel Index, *BSSS* Berlin Social Support Scales, *IES* Impact Events Scale, *SD* standard deviation

*Missing = 1

Logistic regression analyses with the variables that were significant, at *p* < 0.25 in bivariate comparisons in at least one of the two follow-up assessments (see “Methods”), showed that the independent determinants of a greater likelihood of depressive mood at 3 months after stroke were worse functional status, presence of post-traumatic stress symptoms, low perceived social support and no-rt-PA treatment. At 12 months only worse functional status and lower level of education were significant determinants of depression (Table 3).

**Table 3:** Determinants of depression at 3 and 12 months after stroke. Multiple logistic regression analysis

Determinants	Depressive symptoms at 3 months post-stroke		Depressive symptoms at 12 months post-stroke	
	OR (95% CI)	*p*	OR (95% CI)	*p*
BI ≤ 14 at 3 and 6 months, resp.	24.35 (6.60–89.9)	< 0.001	15.78 (3.96–62.8)	< 0.001
IES > 33 at 3 and 6 months, resp.	9.32 (3.17–27.42)	< 0.001	–	
BSSS < Me. at 3 and 6 months, resp.	3.68 (1.38–27.42)	0.009	–	
Stroke treatment—no-rt-PA	3.19 (1.18–8.62)	0.022	–	
Education—primary/basic vocational	–		3.61 (1.36–9.61)	0.010

*NIHSS* National Institutes of Health Stroke Score, *BI* Barthel Index, *BSSS* Berlin Social Support Scales, *IES* Impact Events Scale, *Me*. median, *resp*. respectively, *OR* odds ratio, *CI* confidence interval

Variables included in the multiple regression analyses, *p* < 0.25 in bivariate analyses in at least one of the two assessments

## Discussion

This is one of the very few studies to examine, prospectively, depressive symptoms in thrombolysed stroke patients in comparison to patients who had not received thrombolytic treatment [2, 13]. The two groups were well balanced in terms of several characteristics (see Table 1), except for stroke symptoms which were more pronounced in the rt-PA-treated patients at both admission and discharge. Such a difference may be due to the fact that patients with more severe symptoms reach the hospital early enough to be considered for intravenous therapy [2, 13]. Nevertheless, after 3 months, no significant differences between these groups were seen.

Regarding the first aim of our study, we found that at 3 months post-stroke, the prevalence of depressive symptoms among patient who were, and who were not, thrombolysed was not significantly different. Also, after 1 year, there were no significant differences between the groups. The results obtained are consistent with the study of Weerd et al. [13] who also could not find essential differences in the proportion of depressive disorders at 1 year post-stroke among elderly patients treated and not-treated with rt-PA. Indeed, these findings may suggest that thrombolytic therapy does not have an effect on patients’ emotional state.

On the other hand, it cannot be ruled out that, if thrombolysis had not been used, the early neurological improvement observed in the thrombolysed group in our study would have been lower, and consequently, functional disability, as well as depressive symptoms, would have been more frequent. This speculation might be supported by a recently published study [12], which showed that patients treated with rt-PA less frequently used antidepressants due to newly identified depression (11.3%), than those being matched for baseline NIHSS score treated with standard care alone (19.8%) (hazard ratio = 0.52), although this report should be considered with caution as undertreatment of depression is common in subjects after stroke [31, 32]. It is also noteworthy that in our study the chance of being depressive was found to be lower (although statistically insignificant) in the patients treated with rt-PA after 3 months compared to the non-thrombolysed patients. Admittedly, this may simply reflect the play of chance, due to the relatively small sample size, but it is also possible that it indicates a real trend, possibly related to strategies for coping with psychological distress. For example, there might have been a downward counterfactual thinking [33], i.e. a conviction that it could have been worse if the thrombolysis had not been used. Interestingly, after 12 months this probable trend was in the opposite direction, which may indicate that different, perhaps less favourable, factors might have played a role in this more distant post-treatment period. This issue undoubtedly warrants further research. Hopefully, the ECASS-4: ExTEND study will bring more information on the relationships between thrombolytic treatment and post-stroke depression [34].

The prevalence rates of depressive symptoms in our patients at both follow-ups were substantial although a little lower than the 31% pooled estimate up to 1 year after stroke reported in the most recent meta-analysis by Hackett et al. [35]. The rates in our study were relatively stable between 3 and 12 months after hospitalization, which is in line with several earlier investigations showing a fairly constant frequency of PSD throughout the first year [31, 36, 37]. This confirms the fact that all stroke patients, irrespective of the form of treatment given, require continuous vigilance for the development of depression.

Regarding the second aim of our study, we found that a few factors were independently related to the depressive status and that the set of these factors differed depending on the time elapsed since the stroke. The most important predictor at 3 months post-stroke was functional disability. In this respect, our findings further support the well-known relationships between PSD and impairment of daily living activities [8, 9, 38].

The next factor independently associated with depression was the presence of posttraumatic stress symptoms. As mentioned in “Introduction”, it has been hypothesized that PSD in the thrombolysed patients might develop as a psychological reaction to the traumatic event, namely the stroke itself, additionally reinforced by the exposure to “fast-paced workup of peri-thrombolysis procedures” [14, p. 1852]. From this point of view, thrombolytic therapy, apart from its well-known positive effect, may, paradoxically, also have a negative impact. Our study confirmed the links between posttraumatic stress symptoms and PSD reported by other authors [39–41]. However, in contrast to the above hypothesis, it was the lack of rt-PA treatment, adjusted for functional disability, social support and posttraumatic stress symptoms that turned out to be the factor associated with depressive symptoms. This seems to be in line with what is said above about a lower OR for depression in the rt-PA-treated group and suggests that thrombolysis may play a positive, perhaps indirect, role in relation to PSD.

As regards the findings concerning distress reaction in our sample (25%), they are in agreement with earlier studies reporting an elevated prevalence of posttraumatic stress disorder, not only in stroke patients but also in those after a transient ischaemic attack [42].

Consistent with our expectations and previous reports [43], we found that a low level of perceived social support was the predisposing factor for depressive symptoms at 3 months after stroke onset. Social support is considered as a buffer against the potentially pathogenic influence of stressful events through its contribution to a complex mechanism of stress, reducing the negative consequences of these events and facilitating constructive coping [44].

At 12 months post-stroke, only two factors, among those considered in our study, were independently associated with depressive symptoms, namely worse functional status and education. The role of education in relation to mood disorders is not clear. However, it is possible that individuals with a low level of education are less likely to exhibit help-seeking behaviors [45] or to develop the self-adjustment abilities which can be helpful in accommodating the change [46].

In our work, personal variables, such as gender, age and living arrangement, were not associated with depressive symptoms. This is in agreement with the majority of studies [9], although there are a number of them which have found that sex (female), age (younger/older) and living alone were related to PSD [13, 37, 47, 48]. Also, lesion location was not predictive for depression in our cohort, which is in line with several previous results [9, 10]. However a longstanding debate on the role of this factor is still open [49, 50].

### Limitations

Our study has important limitations. First, we used screening tools (although well validated), rather than structured clinical interviews. The prevalence of depressive symptoms and post-stroke posttraumatic stress symptoms might, therefore, be overestimated. On the other hand, patients with severe aphasia or other severe stroke symptoms dropped out of the study and were not assessed. These patients might have been those more prone to develop depression. For that reason, the frequency of depressive symptoms may have been underestimated. Second, the patients were recruited from one single centre, the sample size was relatively small and the inclusion criteria required patients to be living in their home, all factors which may limit the generalization of the results. Third, there was a significant drop-out rate, due to lost-to-follow-up, which could bias the results.

## Conclusions

(1) Thrombolysed and non-thrombolysed stroke survivors had similar frequency of depressive symptoms although the thrombolysed patients had more severe neurological deficits in the acute phase. It can be assumed that if thrombolysis had not been used, depressive symptoms would have been more frequent. (2) Lack of the rt-PA treatment was associated with three-time greater odds of screening for PSD at 3 months post-stroke, after adjustment for other PSD correlates. (3) Therefore, thrombolytic therapy seems to have a positive, but indirect, effect on patients’ mood, especially in the first months after stroke. (4) All stroke patients, irrespective of the method of treatment, should be monitored for the presence of depression.
